# Models for the indices of thermal comfort

**Published:** 2008-04-15

**Authors:** Streinu-Cercel Adrian, Costoiu Sergiu, Mârza Maria, Streinu-Cercel Anca, Mârza Monica

**Keywords:** management, biomedical engineering, biotechnology, thermal comfort

## Abstract

The current paper propose the analysis and extension formulation required for establishing decision in the management of the medical national system from the point of view of quality and efficiency such as: conceiving models for the indices of thermal comfort, defining the predicted mean vote (on the thermal sensation scale) „PMV”, defining the metabolism „M”, heat transfer between the human body and the environment, defining the predicted percent of dissatisfied people „PPD”, defining all indices of thermal comfort.

## 1. Introduction

In order to analyze and formulate the necessary extensions for implementing the decision in the management of the national’s medical system from the point of view of quality and efficiency, several conditions must be fulfilled and one of the most important is the condition of thermal comfort of the patient.

Human thermal comfort is the state of mind that expresses satisfaction with the surrounding environment, according to ASHRAE Standard 55. Achieving thermal comfort for most occupants of buildings or other enclosures is a goal of HVAC design engineers. Indoor air quality, although not a part of thermal comfort, is a key concern of HVAC designers.

Fundamental studies of thermal comfort, such as acceptable ranges of dry-bulb temperatures, relative humidity, and activity levels were completed in the 1970s. Many of these studies, which led to the development and refinement of ASHRAE Standard 55, were performed at Kansas State University by Ole Fanger and others. Some key findings were that not everyone will be satisfied by a particular set of indoor environmental conditions, but in some ranges of conditions, about 80% express satisfaction. If very good conditions are in place, a maximum of 95% of all persons may be satisfied. Statistical methods were used to evaluate the thermal comfort opinions of the many test subjects to yield what are known as comfort conditions; the predicted mean vote (PMV) was one of the used measures. Most important for thermal comfort is the so called operative temperature. This is the average of air dry-bulb temperature and of the mean radiant temperature at a given place in the room. In addition, there should be low air velocities and no 'drafts', little variation in radiant temperatures from different directions in the room, the humidity has to be in a comfortable range, and the air temperatures at a height of 0.1 m above the floor should not be more than 2°C lower than the temperature at the place of the occupant's head. Also, temperatures should not change too rapidly - neither across space nor in time.

In addition to environmental conditions, thermal comfort depends on clothing and the activity level of a person. The amount of clothing is measured against a standard amount that is roughly equivalent to a typical business suit, shirt, and undergarments. Activity level is compared to being seated quietly, such as in a classroom.

This standard amount of insulation required to keep a resting person warm in a windless room at 70 °F (21.1°C) is equal to one Clo. Clo units can be converted to R-value by multiplying Clo by 0.88 and R-value can be converted to Clo by multiplying R-value by 1.136.

## 2. Models for the thermal comfort indices

Therefore the term “thermal comfort’’ describes a person’s psychological state of mind and is usually referred to in terms of whether someone is feeling too hot or too cold. [**1**]. Thermal comfort is very difficult to define because one needs to take into account a range of environmental and personal factors when deciding what will make people feel comfortable. These factors make up what is known “the human thermal environment’’.

QM−Qdif−Q−evapQL=Q+rQc

(1)

where

- Qm is the heat produced by metabolism;

- Qdif is the lost heat through diffusion of water vapours through skin;

- Qevap is the heat lost through evaporation of perspiration;

- Ql is the latent heat of evaporation of the perspiration

- Qr is the radiant heat lost at the exterior surface of clothing;

- Qc is the heat lost to environment through convective transfer.

Fanger extended the usefulness of his work by proposing a method by which the actual thermal sensation could be predicted. His assumption for this was that the sensation experienced by a person was a function of the physiological strain imposed on him by the environment. This he defined as “the difference between the internal heat production and the heat loss to the actual environment for a man kept at comfort values for skin temperature and sweat production at the actual activity level” (Fanger1970). He calculated this extra load for people involved in climate chamber experiments and plotted their comfort vote against it. Thus he was able to predict what comfort vote would arise from a given set of environmental conditions for a given clothing insulation and metabolic rate. Tables of PMV are available for different environments for given clothing and metabolic rates. Such tables form the basis of ISO standard 7730. To be noted however that his method for PMV is inconsistent with the basic assumptions of his equation (Humphreys and Nicol 1995).

Fanger realised that the vote predicted was only the mean value to be expected from a group of people, and he extended the PMV to predict the proportion of any population who will be dissatisfied with the environment [**2**]. A person's dissatisfaction was defined in terms of his/her comfort vote. Those who voted outside the central three scaling points on the ASHRAE scale were counted as dissatisfied. PPD is defined in terms of the PMV, and adds no information to that already available in PMV. The distribution of PPD is based on observations from climate chamber experiments and not from field measurements.

## 3. Predicted mean vote (PMV)

**Fig. 1:**
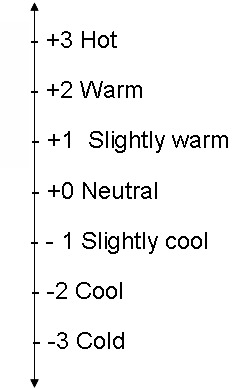
PMV scale

and it is expressed by the following relation:

$$\begin{array}{l} PMV=(0.303\cdot e^{-0.036M}+0.028)\cdot \{(M-W)-3.05\cdot 10^{-3}\cdot \\ \left[5733-6.99\cdot (M-W)-\rho_{a}\right]-0.42\cdot \left[(M-W)-58.15\right]\\ -1.7\cdot 10^{-5}\cdot M\cdot (5867-\rho_{a}-0.0014\cdot M\cdot (34-t_{a})\\ -3.96\cdot 10^{-8}\cdot f_{cl}\cdot \left[{\left(t_{cl}+273\right)}^{4}-{\left(\overset{-}{t_{r}}+273\right)}^{4}\right]-f_{cl}\cdot h_{c}\cdot (t_{cl}-t_{a})\} \end{array}$$

(1)

where:

- M is the metabolic rate (met)

$$1met=58.2\frac{W}{m^{2}}$$

- W is the mechanical work done by the human body

$$\frac{W}{m^{2}}$$

- Icl is the cloth index (clo)

$$1clo=0.155\frac{m^{2}{}^{\circ}C}{W}$$

- fcl is the ratio between the dressed surface of the human body and the undressed surface of the human body

## 4. Energetic metabolism

Our living bodies generate heat because we are homoeothermic (warm-blooded) creatures. The rate at which heat is produced depends primarily on our metabolic rate. The metabolic rate is our ability to generate heat and it is mostly a function of our level of muscular activity. Some of the energy generated by muscular activity will be directly translated into work (force x distance) and the excess energy will be dissipated as heat [**3**].

Because heat exchange with our environment is primarily via the skin, the met unit is defined in terms of both heat energy and surface area. The unit of the metabolic rate is known as “met” which is equivalent to 58.2W/m2. Some values of metabolic rates for various typical activities are presented in **Table 1**.

The normal body core temperature is 37°C, the human body having separate heat- and cold-sensors. The heat sensor is located in the hypothalamus and signals when temperature is higher than 37°C. The cold sensors are located in the skin and send signals when skin temperature is below 34°C [**4**].

**Table 1:** Some values of metabolic rates

Activity	Metabolic rates	
Reclining	46 W/m2	0.8 Met
Seated relaxed	58 W/m2	1.0 Met
Clock and watch repairer	65 W/m2	1.1 Met
Standing relaxed	70 W/m2	1.2 Met
Car driving	80 W/m2	1.4 Met
Standing, light activity (shopping)	93 W/m2	1.6 Met
Walking on the level, 2 km/h	110	1.9 Met
Standing, medium activity (domestiv work)	116	2.0 Met
Washing dishes standing	145	2.5 Met
Walking on the level, 5 km/h	200	3.4 Met
Building industry	275	4.7 Met
Sports - running at 15 km/h	550	9.5 Met

The heating mechanism reduces blood flow and gives shivering sensations while the cooling mechanism increases blood flow and gives sweating sensations (evaporation). The higher the temperature difference, the more impulses are sent, if the impulses are of the same magnitude, we feel thermally neutral and if not, we feel cold or warm. Thermal comfort can only be maintained when heat produced by metabolism equals heat lost from the body.

The equation for general heat balance is given by:

S = M-W-E-Q (2)

where:

- S is the rate of heat storage in the human body

- E is the rate of total evaporation loss

- Q is the rate of total heat loss through skin (dry heat exchange).

The metabolic rate depends on muscular activities, environment, body size, etc. The mechanical work (W) is the energy in human body transformed into external mechanical work. The more physical work we do, the more heat we produce. The more heat we produce, the more heat needs to be lost so we don’t overheat. The impact of metabolic rate on thermal comfort is critical.

When considering these factors, it is also essential to consider a person’s own physical characteristics.

A person's physical characteristic should always be borne in mind when considering their thermal comfort, as factors such as their size and weight, age, fitness level and sex can all have an impact on how they feel, even if other factors such as air temperature, humidity and air velocity are all constant.

The evaporative heat loss (E) is a release of latent heat energy from evaporation of body fluids by two components: respired vapor loss, Eres (respiration heat losses: latent Erel and sensible Erec) and evaporative heat loss from skin Esk (includes skin diffusion Edif and regulatory sweating Ersw). The evaporative heat loss is expressed by the following relation:

$$E=E_{res}+E_{sk}=E_{rel}+E_{rec}+E_{dif}+E_{rsw}$$

(3)

The dry heat exchange is done through convective and radiative heat transfer, the heat loss by radiation is done if skin temperature >the temperature of surrounding surfaces and the heat loss by convection is done if skin temperature > the dry bulb temperature.

The values for the calculation of insulation of clothing in an activity of 58.2 W/m2 (1met) and a relative humidity of 50% are given in **Table 2**.

The ratio between the dressed surface of the human body and the undressed surface of the human body is a factor that has a predefined values function to the cloth index and it is given by:

$$f_{cl}=\left\{ \begin{array}{l} 1.00+1.290\cdot I_{cl}\ldots for\ldots I_{cl}\leq 0.078\frac{m^{2}\cdot {}^{\circ}C}{W}\\ 1.05+0.645\cdot I_{cl}\ldots for\ldots I_{cl}>0.078\frac{m^{2}\cdot {}^{\circ}C}{W} \end{array}\right.$$

(4)

The most important coefficient in heat transfer is the heat transfer coefficient for convection hc and it is given by the next relation:

$$h_{c}=\left\{ \begin{array}{l} 2.38\cdot {\left(t_{cl}-t_{a}\right)}^{0.25}for^{}2.38\cdot {\left(t_{cl}-t_{a}\right)}^{0.25}>12.1\sqrt{v_{ar}}\\ 12.1{\sqrt{v_{ar}}}^{}for^{}2.38\cdot {\left(t_{cl}-t_{a}\right)}^{0.25}<12.1\sqrt{v_{ar}} \end{array}\right.$$

(5)

**Table 2:** Some values for clothes

Garment description		Icl Clo	Icl m^2°C/W
Underwear	Pantyhose	0.02	0.003
	Briefs	0.04	0.006
	Pants long legs	0.10	0.016
Underwear, shirts	Bra	0.01	0.002
	T-shirt	0.09	0.014
	Half-slip, nylon	0.14	0.022
Shirts	Tube top	0.06	0.009
	Short sleeves	0.09	0.029
	Normal, long sleeves	0.25	0.039
Trousers	Shorts	0.06	0.009
	Normal trousers	0.25	0.039
	Overalls	0.28	0.043
Insulated coveralls	Multi-component filling	1.03	0.160
	Fibre-pelt	1.13	0.175
Sweaters	Thin sweater	0.20	0.031
	Normal sweater	0.28	0.043
	Thick sweater	0.35	0.054

where:

- tα is the air temperature °C

- tr is the mean radiant temperature °C

- var is the relative speed of air m/s

- ρα is the partial pressure of water vapours Pa

- tcl is the temperature at the surface of the cloth °C

tcl=35.7−0.028⋅(M−W)−Icl{3.96⋅10−8⋅fcl⋅[(t+cl273)4−(tr−+273)4]+fcl⋅hc⋅(tcl−ta)}

(6)

**Fig. 2:**
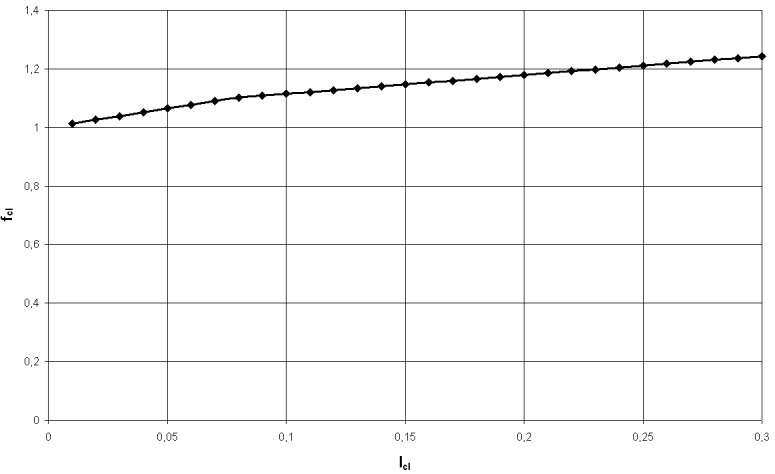
The thermal clothing resistance influence over the clothing factor

**Fig. 3:**
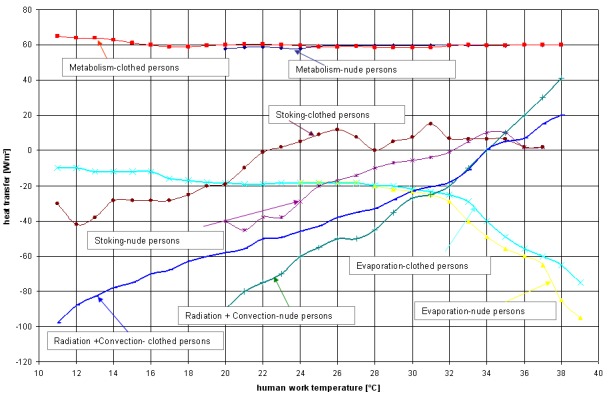
Heat transfer between people and environment

## 5. Predicted percent of dissatisfied people „PPD”

The predicted percent of dissatisfied people is given by the following relation:

$$PPD=100-95\cdot e^{-\left(0.03353\cdot PMV^{4}+0.2179\cdot PMV^{2}\right)}$$

(7)

**Fig. 4:**
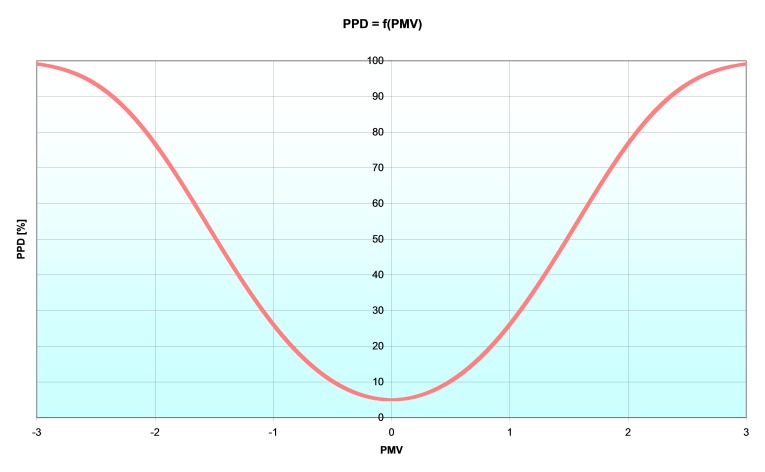
The PPD as a function of PMV

## 6. Thermal comfort indices

***Apparent Temperature (AT)***

Apparent Temperature (AT) (Steadman 1979) quantifies the physiological effects of high heat and high humidity. While AT can easily be calculated as a function of the ambient temperature and moisture, the index includes environmental and physiological variables important in determining human response to environmental stress. These variables include heat generation and loss, fabric resistance, vapour pressure, wind speed, solar radiation, terrestrial radiation, proportion of clothed body, and other factors (Steadman 1984). When constants are the input for these parameters, the index combines temperature and humidity into a single variable. AT is related to the commonly-used heat index in the United States.

***Perceived Temperature (PT)***

The perceived temperature, with units of degrees Celsius, describes a reference environment with fixed parameters in which physiological perception is identical to the experienced environment (Staiger et al.1997) [**5**].

The meteorological variable inputs to PT are air temperature, dew point temperature, wind velocity, total cloud cover, and cloud cover of low, medium and high-level clouds. Because of our lack of specific cloud height information, we developed a parameterization scheme that related surface dew point depression and total cloud amount to the cloud amount at different levels. Further, we performed a sensitivity analysis that demonstrated that the value of PT was much more sensitive to total cloud amount (an observed value that we had available) than to how clouds were divided between the various levels (values that we derived). Therefore, our determination of PT should be mostly unaffected by the limitations of available data.

***Physiological Equivalent Temperature (PET)***

Physiological Equivalent Temperature equates the heat balance of the body in the actual environment to that which is experienced indoors under light activity. PET is the temperature value in degrees Celsius of the indoor environment when the heat balances are identical (Höppe and Mayer 1987; Höppe 1999). PET is calculated from the mean radiative temperature, air temperature, air velocity, and water vapour pressure. Höppe (1999) suggests that a benefit of the PET is that it enables a layperson to make judgments about climate based on personal experience because it is reported in degrees Celsius, whereas other indices do not report a temperature-based value.

***Relative Strain (RS)***

Lee (1979) presents the Belding and Hatch Relative Heat Strain (here referred to as Relative Strain) as an improvement of the former Heat Strain Index. The HSI, in its time, marked a considerable achievement in quantifying the human body’s reaction to heat because it included several important environmental variables as well as the metabolic rate, was based on the physics of heat exchange, and took a relatively simple computational form (Lee 1979). However, the HSI lacked consideration for the resistance of clothing to the loss of both sensible heat and evaporated water vapour, which were included in the refined RS model as calculated by Burton (Lee 1979). RS is “relative” as it is based on standard values for a person performing an established amount of work, with a given rate of ambient air movement. The revision allows the calculation of an RS value for any combination of air temperature, humidity, air movement, activity, radiation load, and clothing insulation (Lee, 1979).

***Spatial Synoptic Classification (SSC)***

The Spatial Synoptic Classification is a site-specific daily discretization of multivariate weather input variables observed diurnally. The resulting classification, developed using discriminated analysis and modified by user knowledge, identifies six primary synoptic weather types described primarily by temperature and moisture, and an additional transition type. In some cases, such as Moist Tropical (warm, moist), additional subcategories are determined to identify extreme days.

Prior research (Kalkstein and Greene 1997) has identified weather situations primarily linked to high mortality in our four city study. The so-called offensive weather types are always associated with high temperatures: Moist Tropical plus (very humid and warm air) and Dry Tropical (warm, dry air). Both synoptic types are deemed offensive in all three cities except for Baltimore, where Dry Tropical is the only one linked to high mortality.

***Standard Effective Temperature (SET) ***

The principles behind the calculation of SET are somewhat similar to those of the PMV. Principally used by the American Society of Heating, Refrigerating and Air-Conditioning Engineers, Inc. (ASHRAE), the SET Index compares individual physiological comfort to a reference environment. The reference environment has a temperature equal to the mean radiant temperature in the ambient environment, wind velocity of zero, and is located at sea level (Ye et al., 2003). 

In addition to these comfort variables, air temperature (T) and dew point temperature (DT) are included to provide low complexity measures for comparison to the more complex indices. Our list of indices is not meant to be exhaustive but was chosen to reflect a portion of the diversity of comfort measures available. AT was calculated according to procedures outlined by Davis et al. (2003). SSC was acquired from a web site monitored by Scott Sheridan at Kent State University (Sheridan, 2002).

The six factors affecting thermal comfort are both environmental and personal. These factors may be independent of each other, but together contribute to a worker’s thermal comfort.

***Environmental factors***

***Air temperature***

This is the temperature of the air surrounding the body. It is usually given in degrees Celsius (°C).

***Radiant temperature***

Thermal radiation is the heat that radiates from a warm object. Radiant heat may be present if there are heat sources in an environment. Radiant temperature has a greater influence than air temperature on how we lose or gain heat to the environment. Our skin absorbs almost as much radiant energy as a matt black object, although this may be reduced by wearing reflective clothing.

***Air velocity***

This describes the speed of air moving across the worker and may help cool him/her if it is cooler than the environment. Air velocity is an important factor in thermal comfort because people are sensitive to it. Still or stagnant air in indoor environments that are artificially heated may cause people to feel stuffy. It may also lead to a build-up of odour. Moving air in warm or humid conditions can increase heat loss through convection without any change in air temperature. Small air movement in cool or cold environments may be perceived as draught. If air temperature is less than skin temperature, it will significantly increase convective heat loss. Physical activity also increases air movement, so air velocity may be corrected to account for a person's level of physical activity.

***Humidity***

If water is heated and evaporates to the surrounding environment, the resulting amount of water in the air will provide humidity. Relative humidity is the ratio between the actual amount of water vapour in the air and the maximum amount of water vapour that the air can hold at that air temperature. Relative humidity between 40% and 70% does not have a major impact on thermal comfort. In some offices, humidity is usually kept between 40-70% because of computers. However, in workplaces which are not air conditioned or where the climatic conditions outdoors may influence the indoor thermal environment, relative humidity may be higher than 70% on warm or hot humid days. Humidity in indoor environments can vary greatly, and may be dependent on whether there are any drying processes (paper mills, laundry etc) where steam is given off. High humidity environments have a lot of vapour in the air, which prevents the evaporation of sweat from the skin. In hot environments, humidity is important because less sweat evaporates when humidity is high (80% +). The evaporation of sweat is the main method of heat loss in humans. When vapour-impermeable PPE is worn, the humidity inside the garment increases as the wearer sweats because the sweat cannot evaporate. If an employee is wearing this type of PPE (e.g. asbestos or chemical protection suits etc) the humidity within the microclimate of the garment may be high.

***Personal factors***

***Clothing insulation***

Clothing, by its very nature, interferes with our ability to lose heat to the environment. Thermal comfort is very much dependent on the insulating effect of clothing on the wearer. Wearing too much clothing or personal protective equipment (PPE) may be a primary cause of heat stress even if the environment is not considered warm or hot. If clothing does not provide enough insulation, the wearer may be at risk from cold injuries such as frostbite or hypothermia in cold conditions.

Clothing is both a potential cause of thermal discomfort as well as a control for it as we adapt to the climate in which we live and play. You may add layers of clothing if you feel cold or remove layers of clothing if you feel warm. However, many companies remove for their employees this ability to make reasonable adaptations to their clothing. It is important to identify how clothing may contribute to thermal comfort or discomfort. It may also be necessary to evaluate the level of protection that any PPE is providing – can less or other PPE be used?

***Work rate/metabolic heat***

The work or metabolic rate is essential for a thermal risk assessment. It describes the heat that we produce inside our bodies as we carry out physical activity. The more physical work we do the more heat we produce. The more heat we produce, the more heat needs to be lost so we don’t overheat. The impact of metabolic rate on thermal comfort is critical. When considering these factors, it is also essential to consider a persons own physical characteristics. A person's physical characteristic should always be borne in mind when considering his/her thermal comfort, as factors such as size, weight, age, fitness level and sex can all have an impact on how he/she feels, even if other factors such as air temperature, humidity and air velocity are all constant

## 7. Conclusions

Hospitals and other healthcare facilities are complex environments that require a special HVAC system design to achieve comfort and to control hazardous emissions [**6**]. There is a great challenge to build an effective HVAC system design to enhance air quality in healthcare facilities. The HVAC system is used to provide the required comfort level and in some cases for healing. Indoor Air Quality (IAQ) is more critical in healthcare facilities due to the presence of hazardous microbial and chemical agents and the increased susceptibility of the patients. Optimum Indoor Air Quality (IAQ) level is the responsibility of architects and mechanical engineers. Hospital air conditioning resumes a more important role than just the promotion of comfort. In many cases, proper air conditioning is a factor in patient therapy; in some instances, it is the major treatment. Studies show that a patient in controlled environments generally has a more rapid physical improvement than have those in uncontrolled environments. Although proper air conditioning designs are helpful for the prevention and treatment of diseases, the application of air conditioning to health facilities presents many specific problems. These are not encountered in conventional comfort conditioning design. Air conditioning, therefore, includes the entire heat exchange operation as well as the regulation of velocity, thermal radiation and quality of air, as well as the removal of foreign particles and vapours.

## 8. Acknowledgments

1. Ministry of Education, Research and Youth.

2. Medical Scientific Academy of Romania.

3. VIASAN PROGRAMME.
